# Comparative Analysis of Bioactive Compounds and Flavor Characteristics in Red Fermentation of Waxy and Non-Waxy Millet Varieties

**DOI:** 10.3390/foods14152692

**Published:** 2025-07-30

**Authors:** Zehui Yang, Jie Liu, Xiaopeng Li, Changyu Zhang, Pengliang Li, Yawei Zhu, Jingke Liu, Bin Liu

**Affiliations:** 1 College of Food Science and Engineering, Northwest A&F University, 22 Xinong Road, Yangling 712100, China; 2 Institute of Biotechnology and Food Science, Hebei Academy of Agriculture and Forestry Sciences, 598 Heping West Road, Xinhua District, Shijiazhuang 050031, China; 3 Integrated Supervision and Service Centre of Hebei Provincial Health Commission, 428 Heping West Road, Xinhua District, Shijiazhuang 050031, China

**Keywords:** “*Red Ferment*”, millet, solid state fermentation, bioactive compounds, volatile compounds

## Abstract

(1) Background: This study investigated changes in bioactive components and volatile compounds (VCs) during the production of red millet by comparing two varieties: Miao Xiang glutinous millet (waxy) and Jigu-42 (non-waxy). The samples were solid-state-fermented with “*Red Ferment*” and evaluated for bioactive components. (2) Methods: Multiple analytical methods—including principal component analysis (PCA), partial least squares-discriminant analysis (PLS-DA) and orthogonal PLS-DA (OPLS-DA), cluster analysis, and correlation analysis—were employed to systematically compare bioactive components and VCs. (3) Results: Significant varietal differences were observed: (1) Miao Xiang glutinous millet showed higher monacolin K (MK) and fatty acid contents; (2) Jigu-42 contained significantly more polyphenols; (3) linoleic acid dominated the fatty acid profiles of two varieties; and (4) a total of twenty-seven VCs were identified, including six alcohols, four aldehydes, seven ketones (corrected from duplicated count), two aromatic hydrocarbons, three heterocycles, one acid, three furans, and one ether. (4) Conclusions: The two varieties exhibited significant differences in MK, pigment profiles, fatty acid composition, polyphenol content, and volatile-compound profiles. These findings provide scientific guidance for the selection of the appropriate millet varieties in functional food production.

## 1. Introduction

Millet, one of the oldest cultivated plants in China, is considered a crop crucial for future food security [1]. It is nutrient dense, gluten free and has excellent nutritional properties, including vitamins, dietary fiber, fatty acids, proteins, and various bioactive constituents and minerals [2]. These components have beneficial effects on blood sugar and cholesterol regulation [3]. Millet plays a role in controlling blood pressure and thyroid function, while its consumption enhances immunity and general health [4]. Consequently, the development of novel functional foods from millet has become an emerging area in the food industry.

Given millet’s exceptional nutritional profile, its potential as a fermentation substrate warrants exploration—particularly when combined with traditional fermentation techniques like those used in red yeast rice (RYR) production. RYR is produced by fermenting rice with *Monascus* spp. [5,6], which can synthesize bioactive metabolites such as pigments [7] and MK [8]. Due to its antioxidant, anti-inflammatory, and potentially medicinal properties [9], *Monascus* is widely used in dietary supplements and nutraceuticals. Additionally, *Rhodotorul* spp. can produce valuable carotenoids and microbial lipids [10], and may enhance the nutritional and flavor profiles of fermented products like fruit juices [11].

Research has demonstrated that millet can serve as an efficient substrate for MK production through *Monascus* solid-state fermentation, showing superior performance compared to traditional rice substrates [12]. Considering the dual benefits of MK and millet nutrition, combining the two systems appears promising. Millet provides higher starch bioavailability for microbial growth, smaller particle size for improved fermentation efficiency, and richer bioactive compounds compared to rice. Furthermore, solid-state fermentation offers significant advantages in MK production, compared to submerged liquid fermentation. It promotes better cell growth, enhances cell membrane fluidity and permeability, and accelerates MK biosynthesis [13]. However, there is still a lack of systematic comparison of millet varieties used for *Monascus* fermentation, especially in terms of bioactive-compound dynamics.

Despite millet’s potential, no studies have systematically compared red fermentation performance across millet varieties, particularly in terms of dynamic metabolite profiling. Therefore, this study systematically evaluated the solid-state fermentation of two millet varieties using red fermentation, with the aim of comparing MK and *Monascus* pigments (MPs) relative to production efficiency and characterizing the dynamic changes of the bioactive and volatile compounds varietal differences between Jigu-42 and Miao Xiang glutinous millet—addressing critical gaps in the current research.

## 2. Materials and Methods

### 2.1. Microorganism and Materials

This study utilized two millet varieties: the variety of waxy foxtail millet is Miao Xiang glutinous millet, produced in Guizhou Province, China, while the variety of non-waxy foxtail millet is Jigu-42, produced in Hebei Province, China. The microbial starter “*Red Ferment*”—a red-pigmented consortium containing *Rhodotorula rubra* (syn. *Rhodotorula ruber* CICC 1235) and *Monascus purpureus* (ACCC 30352)—was purchased from Shandong Hezhong Kangyuan Biotechnology Co. (Zibo, China). Additionally, MK standard (CAS No. 75330-75-5) was procured from TanMo Reference Materials Co., Ltd. (Changzhou, China).

### 2.2. Solid-State Fermentation

Approximately 1.5 kg of each millet variety was soaked in distilled water for 4.5 h, steamed at 100 °C for 30 min until the grains were fully softened, and cooled to room temperature. The cooked millet was mixed with 0.4% (*w*/*w*) “*Red Ferment*” inoculum and 20% (*w*/*w*) distilled water and then placed in static incubation at 35 °C for 14 days. During fermentation, 80 g samples were collected every 2 days, freeze-dried (LABCONCO FreeZone 2.5 L, −50 °C, 24 h, Kansas City, MO, USA), and ground into a fine powder, using a laboratory grinder (FSJ-A03E1, Bear Electric Appliance Co., Ltd., Foshan, China), through an 80-mesh sieve for subsequent analysis.

### 2.3. Determination of Chromatic Characteristics

The color of each fermented sample was measured using a high-precision colorimeter (WSF, Shanghai Precision Science Instrument Co., Ltd., Shanghai, China) and calibrated with white and black tiles, following the method of Viscarra et al. [14]. We performed calibration using a standard whiteboard (100% reflectance) and blackboard (0% reflectance) under D65/10° illuminant conditions. L* represents lightness, while a* indicates redness (positive a*) or greenness (negative a*), and b* indicates yellowness (positive b*) or blueness (negative b*). Whiteness (W) and total color difference (ΔE) were calculated as follows:

(1) $$W=100-\sqrt{{\left(100\;-L^{*}\right)}^{2}+{a^{*}}^{2}+{b^{*}}^{2}}$$

(2) $$∆E=\sqrt{{\left(L^{*}-L_{0}\right)}^{2}+{\left(a^{*}-a_{0}\right)}^{2}+({b^{*}-b_{0})}^{2}}$$

where L_0_, a_0_, and b_0_ represent the respective color values of the unfermented control samples.

### 2.4. Determination of Bioactive Components

#### 2.4.1. Determination of MK

MK in each fermented sample was extracted using 75% ethanol (*v*/*v*) and quantified by a high-performance liquid chromatography (HPLC) device (LC-16, Shimadzu Management (China) Co., Ltd., Pudong District, Shanghai, China) equipped with UV detection, with a standard curve established using lovastatin standards. Briefly, 0.5 g of red millet powder was ultrasonicated in 20 mL of 75% ethanol for 1 h. After centrifugation at 4000 rpm for 10 min, the supernatant was filtered through a 0.22 μm membrane. A 10 μL aliquot of the filtrate was injected into a C18 column (4.6 mm × 250 mm, 5 μm) at a flow rate of 1.0 mL/min, with detection at 238 nm. The column temperature was 35 °C. The mobile phase consisted of eluent A (0.1% [*v*/*v*] phosphoric acid in deionized water) and eluent B (chromatography-grade acetonitrile). The gradient elution program was as follows: 0 min (45% A), 5 min (30% A), 10 min (10% A), 20 min (20% A), and 30 min (45% A).

#### 2.4.2. Determination of MP Content

The yellow, orange, and red pigments were extracted following the same protocol used for MK, and quantified by UV-Vis spectrophotometry at their characteristic absorption wavelengths: 385 nm (yellow), 475 nm (orange), and 505 nm (red). The corresponding color values were calculated using the following equation:

(3) $$color\;values\;(U/g)=\frac{A\times DF\times V}{W}$$

where A is the absorbance of diluent, DF is the dilution factor, V is the volume of pipetted ethanol (mL), and W is the weight of the sample (g).

#### 2.4.3. Determination of Total and Free Fatty Acid Content

The fatty acid analysis was performed according to the method described by Li et al. [15], using an Agilent 7820A gas chromatography (GC) device (Agilent Technologies, Santa Clara, CA, USA) equipped with a flame ionization detector (FID) and automatic injector. The column was an HP-88 (100 m × 0.25 mm i.d., 0.20 μm film thickness, Agilent Technologies, Santa Clara, CA, USA), and the injector temperature was 240 °C. The temperature program followed the following sequence: the initial temperature of the column oven was 130 °C, and it was increased to 240 °C at a rate of 4 °C/min across a duration of 20 min.

#### 2.4.4. Determination of Polyphenols and Flavonoids Contents

Polyphenols and flavonoids were extracted using 70% (*v*/*v*) ethanol. The total polyphenol content (TPC) was determined using the Folin–Ciocalteu method, with absorbance measured at 760 nm. A standard curve created using gallic acid standard (y = 0.0087x + 0.0052, R^2^ = 0.999) was used for the calculation. The total flavonoids content (TFC) was analyzed using the NaNO_2_-Al(NO_3_)_3_-NaOH method through spectrophotometry, with quantification against a rutin standard (≥98% purity, Macklin) calibration curve (y = 0.0094x − 0.0001, R^2^ = 0.999).

### 2.5. HS-SPME-GC–MS

#### 2.5.1. Sample Preparation

Each sample (1 g) was transferred to a separate headspace bottle and mixed with 10 μL of methyl heptanoate (10 µg/mL) as the internal standard. The mixture was extracted using the automatic headspace solid phase microextraction (HS-SPME) and analyzed by using a Thermo Scientific™ TRACE 1310 GC-ISQ 7000 MS system (Thermo Fisher Scientific, Waltham, MA, USA).

#### 2.5.2. GC-MS Conditions

The GC-MS analytical parameters were consistent with previous studies. For SPME-Arrow, the extracted fiber was inserted into the injector port operated in splitless mode. Samples were analyzed on a DB-Wax column (60 m × 0.25 mm i.d. × 0.25 μm film thickness), and the injector port was held at 230 °C. High-purity helium (99.99%) was used as the carrier gas at a constant flow rate of 1.0 mL/min. The oven temperature program consisted of the following: (1) initial hold at 40 °C for 3 min, and (2) a ramp at 4 °C/min to 230 °C. The mass spectrometer was operated in electron impact (EI) mode at 70 eV ionization energy, with the ion source temperature set at 230 °C. Full-scan acquisition was used in the 30 to 400 *m*/*z* range. All analyses were performed in triplicate.

#### 2.5.3. Qualitative Analysis

The detected VCs were identified by comparing their mass spectra with the NIST 05 Standard Spectral Library (match factor > 80%). The retention index (RI) of each volatile substance was calculated from n-alkanes (C_8_–C_20_) and compared with the MS spectra and the pertinent literature to characterize the volatile components.

### 2.6. Electronic-Tongue (E-Tongue) Analysis

Detection was performed using an Astree e-tongue (Alpha Mos, Toulouse, France) equipped with nine cross-selective sensors: two general sensors and seven specific sensors for sour taste, salty taste, umami taste, rich taste, astringency, sweet taste, and bitter taste, respectively [16]. For each sample, the centrifuged fermentation broth was injected into a 25 mL specialized beaker for the e-tongue analysis. The electrode response signal was automatically recorded by the machine every second, and the detection time was 120 s. The average value of 110–120 s was taken as the characteristic value. All samples were tested at a temperature of 25 ± 3 °C.

### 2.7. Statistical Analysis

The data obtained were analyzed using SPSS Statistics 19 (IBM Corp., Armonk, NY, USA) and presented as mean ± standard deviation (SD). Statistically significant differences among the groups were determined by one-way analysis of variance (ANOVA) followed by LSD post hoc tests when uniform variance of the result was identified, or followed by Dunnett’s T3 tests. Statistical significance was set at *p* < 0.05. Origin 2022 was used to visualize the physicochemical parameters, radar maps, and heatmaps. Intergroup correlations were analyzed, and the correlation heatmap was generated using ChiPlot (https://www.chiplot.online/) (accessed on 7 June 2025). PCA PLS-DA and OPLS-DA were performed using SIMCA 14.1 (V16.0.2, Sartorius Stedim Data Analytics AB, Umeå, Sweden).

## 3. Results

### 3.1. Analysis of Chromatic Characteristics

The color parameters of Miao Xiang glutinous millet and Jigu-42 millet during fermentation are shown in Table 1. The L* values of Miao Xiang glutinous millet decreased significantly from 91.53 to 81.18, indicating a decrease in brightness, while Jigu-42 remained stable (91.80 to 91.12). The darker appearance of Miao Xiang glutinous millet was due to its higher pigment accumulation compared to Jigu-42. In Miao Xiang glutinous millet, a* values increased from −2.37 (green) to 19.32 (red), and b* values increased from 24.69 to 52.17 (yellow intensified). In contrast, Jigu-42 showed minimal changes: a* from −2.25 to 0.05 (slight reddening) and b* from 21.94 to 23.02 (stable yellow). The observed changes in color parameters (L*, a*, b*) were closely associated with the synthesis of carotenoids and MPs by *Rhodotorula rubra* and *Monascus* throughout the fermentation process [17]. Additionally, Miao Xiang glutinous millet’s whiteness (W) dropped significantly from 73.79 to 41.27.

### 3.2. Bioactive Components

#### 3.2.1. Analysis of Changes in the MP Contents

**Figure 1:**
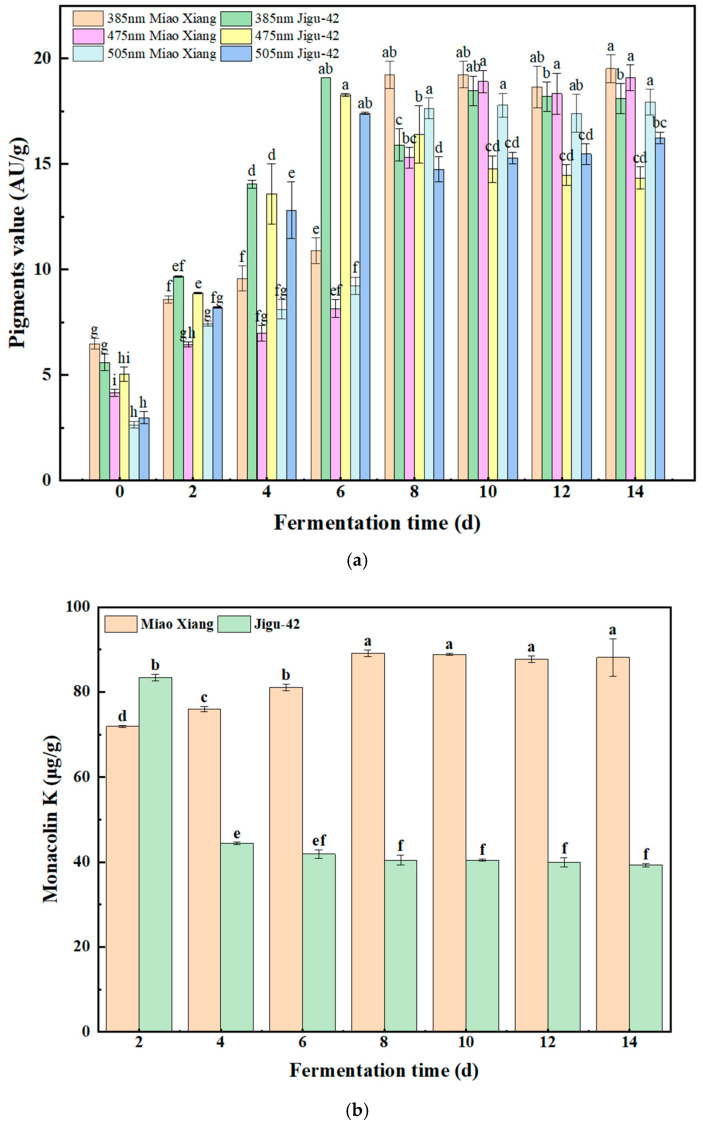

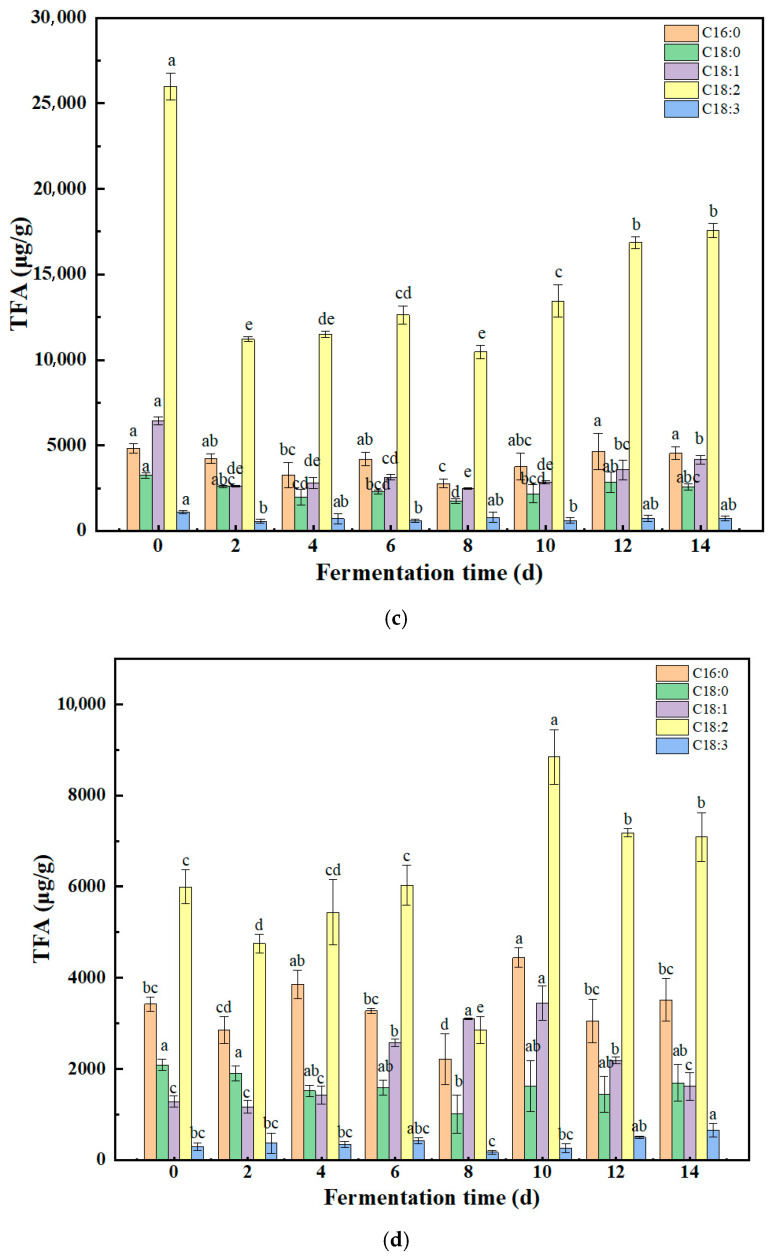

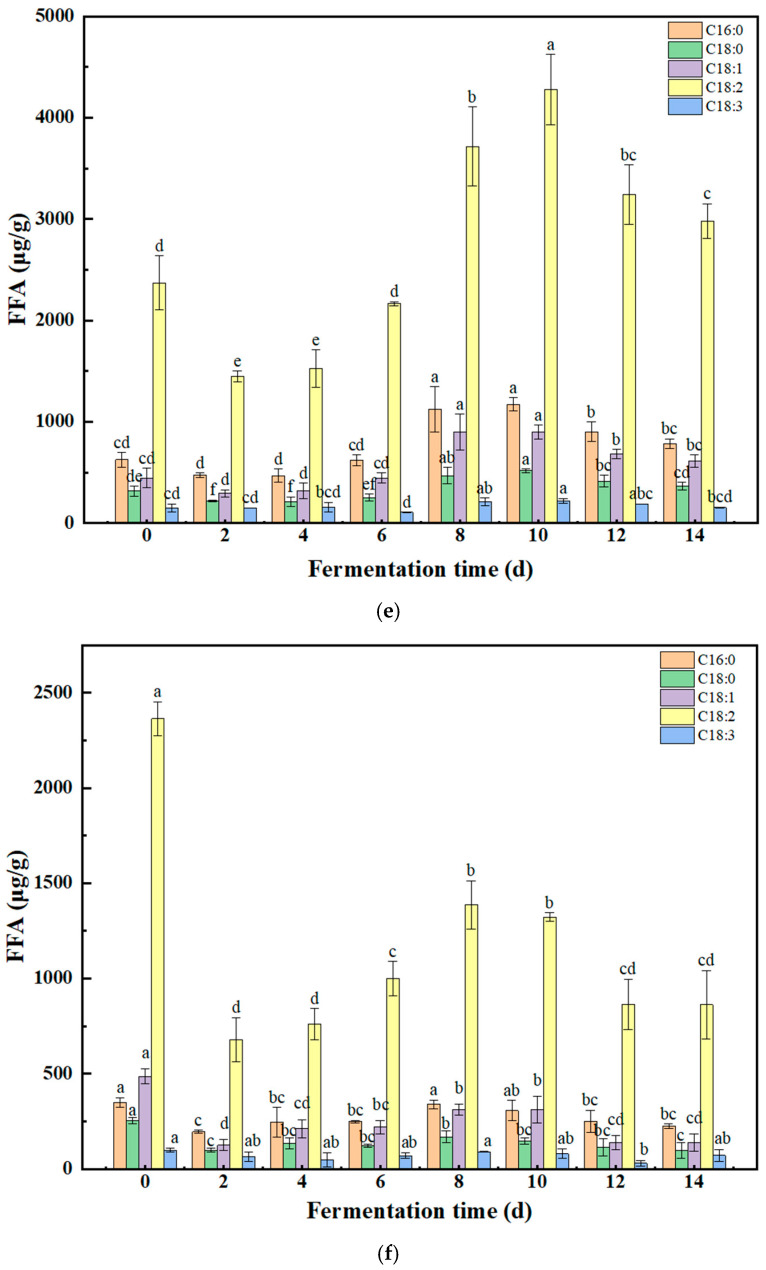

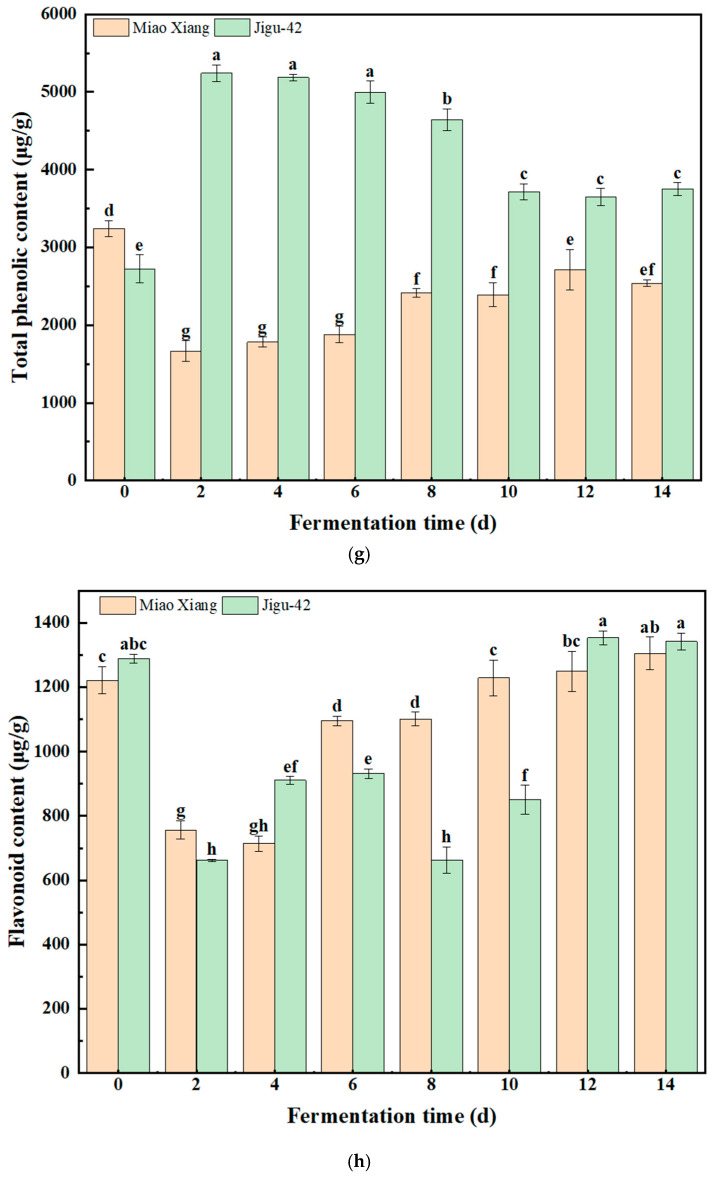
The bioactive-component contents during millet fermentation: (**a**) MPs; (**b**) MK; (**c**) Miao Xiang glutinous millet—total fatty acids; (**d**) Jigu-42—total fatty acids; (**e**) Miao Xiang glutinous millet—free fatty acids; (**f**) Jigu-42—free fatty acids; (**g**) polyphenols; and (**h**) flavonoids. Different letters in the figures indicate significant differences (*p* < 0.05, one-way ANOVA followed by Duncan’s multiple range test.

The bioactive component contents of Miao Xiang glutinous millet and Jigu-42 millet are presented in Figure 1. Figure 1a demonstrates significant differences in yellow, orange, and red pigment production between the Miao Xiang glutinous millet and Jigu-42 groups throughout the fermentation period. The contents of all three pigments increased significantly after fermentation. In the Miao Xiang glutinous millet group, the yellow, orange, and red pigments reached their highest levels on day 14, with values of 19.52 AU/g, 19.09 AU/g, and 17.93 AU/g, respectively. These values represented increases of 200.6%, 360.4%, and 575.9% compared to the unfermented samples. In the Jigu-42 group, the highest pigment levels were observed on day 6, with levels reaching 19.08 AU/g (yellow), 18.28 AU/g (orange), and 17.40 AU/g (red), which were 240.7%, 262.7%, and 485.3% higher than those in the unfermented group, respectively.

The pigment content produced by the Miao Xiang glutinous millet group after fermentation was higher than that of the Jigu-42 group. This difference can be attributed to the higher branched-chain starch content in Miao Xiang glutinous millet [18], which enhances water absorption and retention [19]. These properties significantly promote MP production and increase α-amylase activity [20]. MPs exhibit notable α-glucosidase inhibitory activity, contributing to hypoglycemic effects; this property suggests potential applications in type 2 diabetes management [21]. MP derivatives were isolated from highland barley *Monascus* for the first time, some with moderate inhibitory effects on pancreatic lipase activity and some with certain hepatoprotective activities [22]. Recent studies also show that injectable adhesive hydrogels containing MP nanoparticles can reduce oxidative stress in cardiomyocytes and decrease cardiomyocyte apoptosis [23].

#### 3.2.2. Analysis of the Change in MK Content

The MK content is presented in Figure 1b. The MK content in Miao Xiang glutinous millet increased significantly with fermentation time and stabilized during the intermediate and late fermentation phases. In contrast, the Jigu-42 millet showed a progressive decline in MK content throughout the fermentation period. On day 8 of fermentation, Miao Xiang glutinous millet reached its peak MK concentration (89.15 ± 0.76 µg/g), while Jigu-42′s highest MK concentration (83.42 ± 0.74 µg/g) was observed on day 2.

#### 3.2.3. Analysis of the Change in Fatty Acid Content

Millet is rich in fatty acids, with five major fatty acids (C16:0–C18:3) identified in both the Miao Xiang glutinous millet and the Jigu-42 varieties. Linoleic acid (C18:2) was the predominant fatty acid, constituting 70% of the total fatty acid (TFA) content [24]. As shown in Figure 1c,d, the TFA content in Miao Xiang glutinous millet decreased significantly with prolonged fermentation. In contrast, only stearic acid (C18:0) content decreased in Jigu-42 millet. Notably, Jigu-42 reached peak concentrations of three fatty acids on day 10 of fermentation: palmitic acid (C16:0), oleic acid (C18:1), and linoleic acid (C18:2). These contents were 4446.84 µg/g, 3443.34 µg/g, and 8845.62 µg/g, respectively, which were 29.8%, 167.6%, and 47.6% higher than the unfermented group. These observations align with recent findings that most medium and long chain fatty acids show significant negative correlations with straight-chain starch content [25], which can be used to elucidate the reason why Jigu-42 maintained lower TFA levels than Miao Xiang glutinous millet. The TFA content of the Miao Xiang glutinous millet group decreased after fermentation, which may stem from the microbial metabolism of the *Rhodotorula rubra* and *Monascus* spp. or from their conversion to the other VCs that contribute to the flavor of *Monascus*-fermented millet [26].

Figure 1e,f present the dynamics of free fatty acid (FFA) content during fermentation in both millet varieties. By fermentation day 10, the Miao Xiang glutinous millet group showed significant increases in five principal free fatty acids (FFAs): palmitic acid (C16:0), stearic acid (C18:0), oleic acid (C18:1), linoleic acid (C18:2), and linolenic acid (C18:3). The content levels were 1172.62 µg/g, 515.89 µg/g, 898.36 µg/g, 4279.01 µg/g, and 220.31 µg/g, respectively. These values increased by 87.6%, 63.8%, 101.9%, 80.5%, and 45.6%, respectively, compared to the unfermented control group.

In contrast, the Jigu-42 group exhibited a progressive decline in FFA content with extended fermentation time. Studies have demonstrated that millet fatty acids possess hypoglycemic properties due to their ability to maintain low starch digestibility, with oleic acid (C18:1) being particularly effective in reducing starch hydrolysis rates [27].

#### 3.2.4. Analysis of the Changes in the Levels of Polyphenols and Flavonoids

The total polyphenol content (Figure 1g) of fermented Miao Xiang glutinous millet was lower than that of the unfermented control, whereas fermented Jigu-42 exhibited a higher content than its unfermented counterpart. In raw Jigu-42 millet, the total polyphenol content was 2725.52 µg/g, peaking at 5242.76 µg/g on day 2 of fermentation, representing a 92.36% increase.

The flavonoid content (Figure 1h) increased after 10 days of fermentation, with Miao Xiang glutinous millet exhibiting lower levels than Jigu-42. The initial values in raw millet were 1221.70 µg/g for Miao Xiang glutinous millet and 1288.37 µg/g for Jigu-42. The maximum content was observed on day 14 for Miao Xiang glutinous millet (1304.68 µg/g) and day 12 for Jigu-42 (1353.62 µg/g), representing increases of 6.79% and 5.06%, respectively. Millet shows potential as a healthy food material and a natural antioxidant source.

The increased polyphenol content after fermentation can be attributed to a few factors: (1) enhanced activity and bioavailability of polyphenol-related enzymes during fermentation [28], and (2) acidification in solid-state fermentation that promotes hydrogen ion oxidation and phenolic structure rearrangement [29]. Dietary intake of polyphenol-rich foods has been shown to stimulate the growth of beneficial gut microbiota [28] while inhibiting pathogenic bacteria proliferation [30].

### 3.3. Characterization of VCs

**Figure 2:**
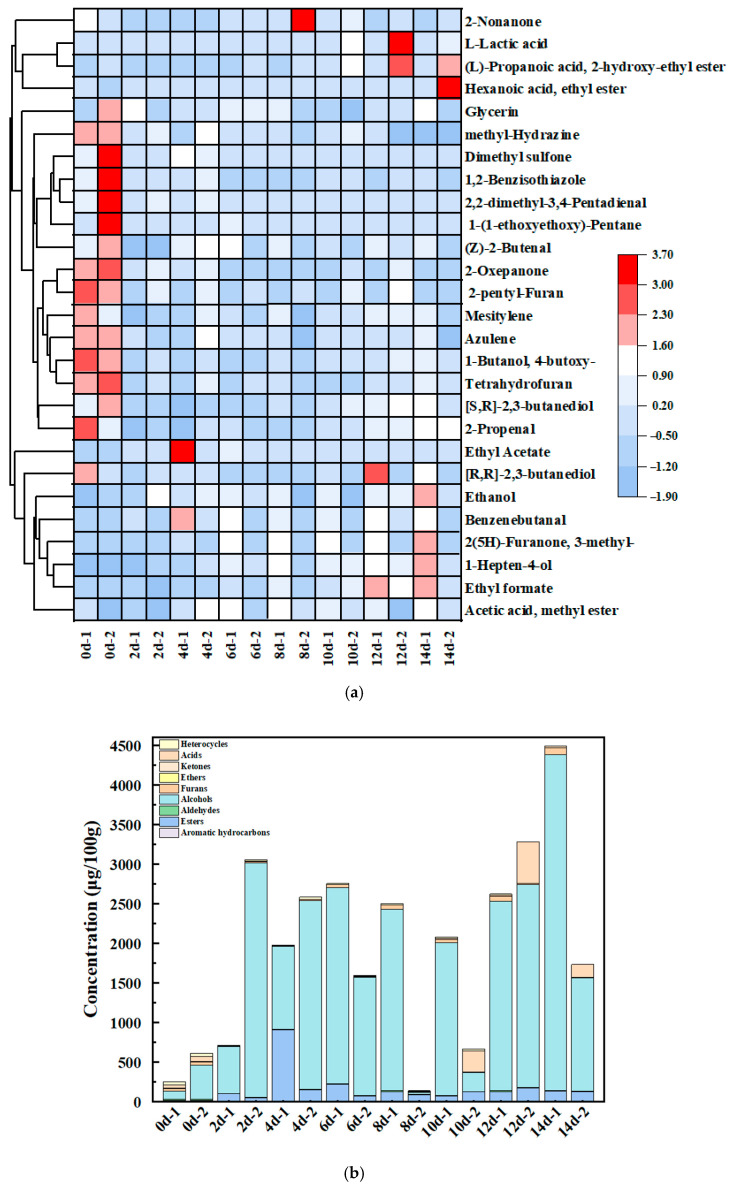

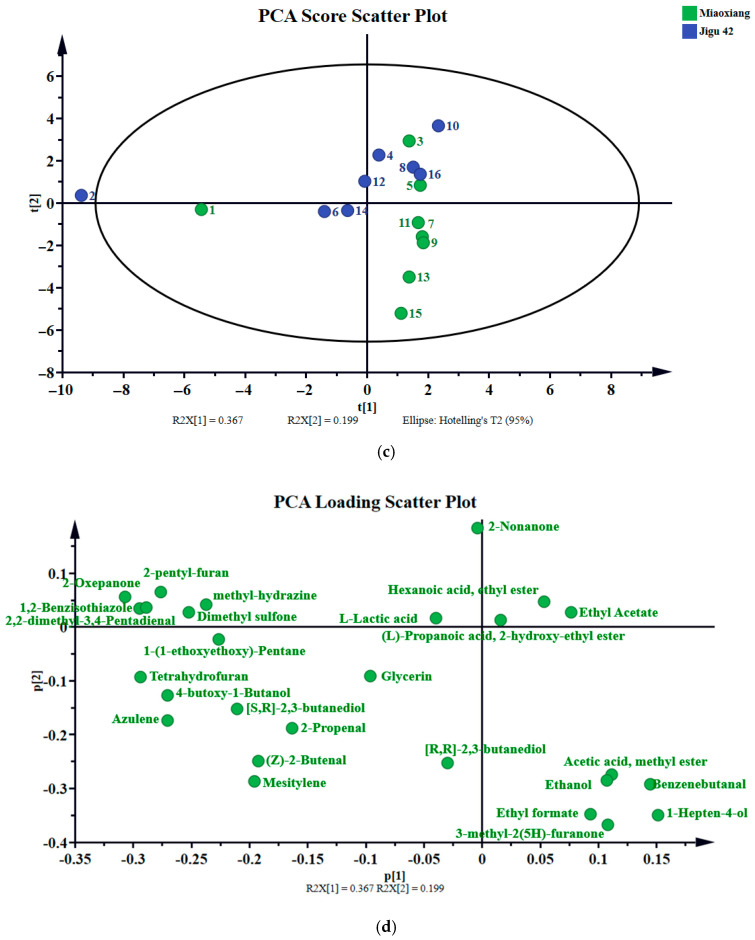

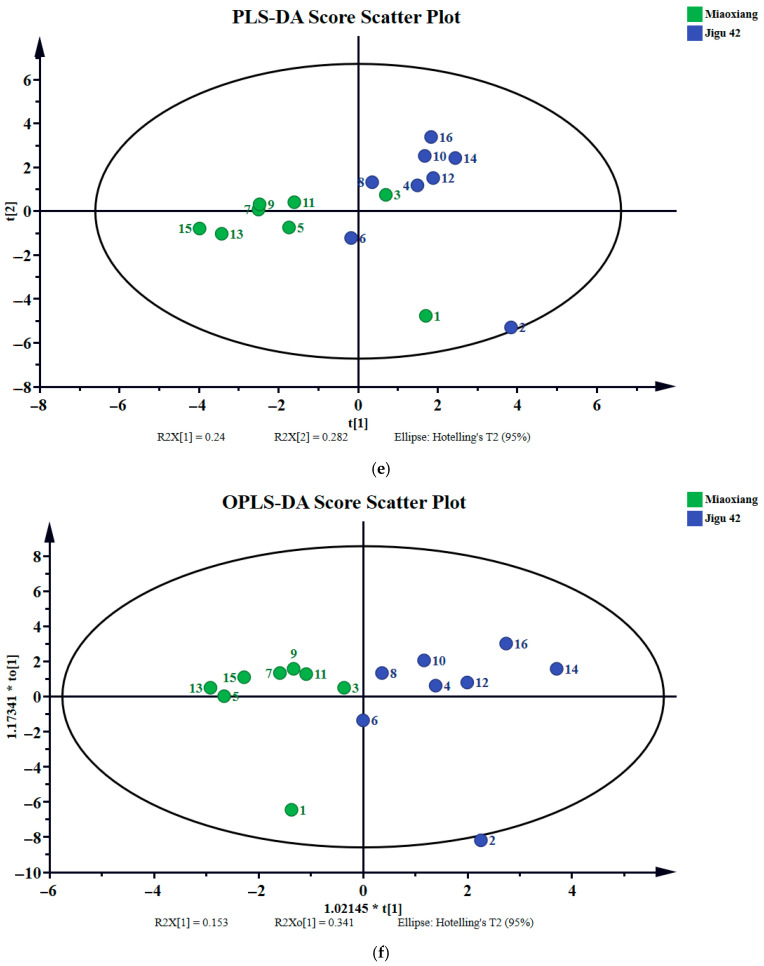

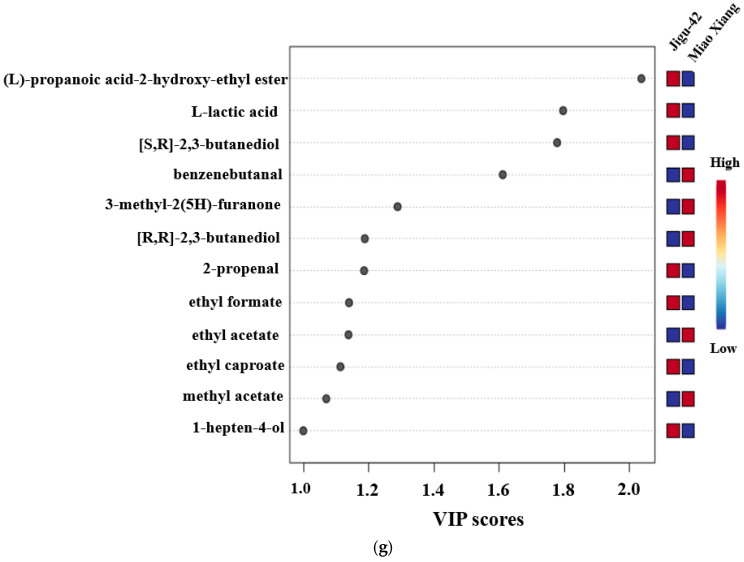
Analysis of VCs in millet varieties: (**a**) heat map of VCs across samples (1: Miao Xiang glutinous millet; 2: Jigu-42); (**b**) relative abundances of dominant VCs (1: Miao Xiang millet; 2: Jigu-42; data are mean ± SD, unit: μg/100 g); (**c**) PCA score scatter plot; (**d**) PCA loading scatter plot; (**e**) PLS-DA score scatter plot; (**f**) OPLS-DA score scatter plot; and (**g**) VIP scores from PLS-DA (VIP > 1).

To investigate the evolution of aroma profiles in “*Red Ferment*”-fermented millet during the 14-day fermentation period, VCs were analyzed at 2-day intervals (days 0, 2, 4, 6, 8, 10, 12, and 14) using SPME-Arrow-GC-MS. Cluster analysis (Figure 2) revealed significant differences in flavor profiles among different fermentation periods and millet varieties, as visualized in the heatmap (Figure 2a,b). The 27 identified VCs were classified into nine groups: six alcohols, six esters, four aldehydes, one ketone, two aromatic hydrocarbons, three heterocycles, one acid, three furans, and one ether.

The 27 identified VCs in “*Red Ferment*”-fermented millet were predominantly alcohols and esters (Table S1). Among the six identified alcohol compounds, ethanol was the most abundant, followed by 1-hepten-4-ol, 4-butoxy-1-butanol, [S, R]-2,3-butanediol, glycerin, and [R, R]-2,3-butanediol. Notably, sugar serves as the primary carbon source for the growth and metabolism of *M. purpureus* during fermentation [13,31]. Six ester compounds were identified: methyl acetate, ethyl formate, ethyl acetate, ethyl hexanoate, 2-hydroxyethyl propanoate, and ε-caprolactone (syn. 2-oxepanone). Ethyl esters (particularly ethyl acetate) constituted the dominant subgroup, which is known to impart milky, fruity, and sweet aroma notes to fermented millet [32].

The alcohol content in Miao Xiang glutinous millet increased significantly during fermentation, peaking on day 14, whereas Jigu-42 millet reached its maximum ester content on day 4 (Figure 2b). During the later fermentation stages, L-lactic acid accumulation was observed, a phenomenon which may contribute to rancid off-flavors. On day 8, the volatile-compound content of Jigu-42 millet was at its most minimal point. In addition, we performed multivariate statistical analyses of the VCs of Miao Xiang glutinous millet and Jigu-42 using PCA, PLS-DA, and OPLS-DA. The results indicated that fermentation significantly affected millet odor profiles, with marked differences between the two varieties (Figure 2c–f).

PCA scores and loading plots revealed that methyl acetate, ethanol, ethyl formate, [R, R]-2,3-butanediol, 3-methyl-2(5H)-furanone, 1-hepten-4-ol, and benzenebutanal were more abundant in Miao Xiang glutinous millet, whereas 2-nonanone, L-lactic acid, (L)-propanoic acid-2-hydroxy-ethyl ester, glycerin, and hydrazine-methyl were enriched in Jigu-42 (Figure 2c,d). Based on PLS-DA, twelve discriminatory VCs (VIP > 1) were identified: 1-hepten-4-ol, methyl acetate, ethyl caproate, ethyl acetate, ethyl formate, 2-propenal, [R, R]-2,3-butanediol, 3-methyl-2(5H)-furanone, benzenebutanal, [S, R]-2,3-butanediol, L-lactic acid, and (L)-propanoic acid-2-hydroxy-ethyl ester (Figure 2g).

### 3.4. Analysis Using the E-Tongue

**Figure 3:**
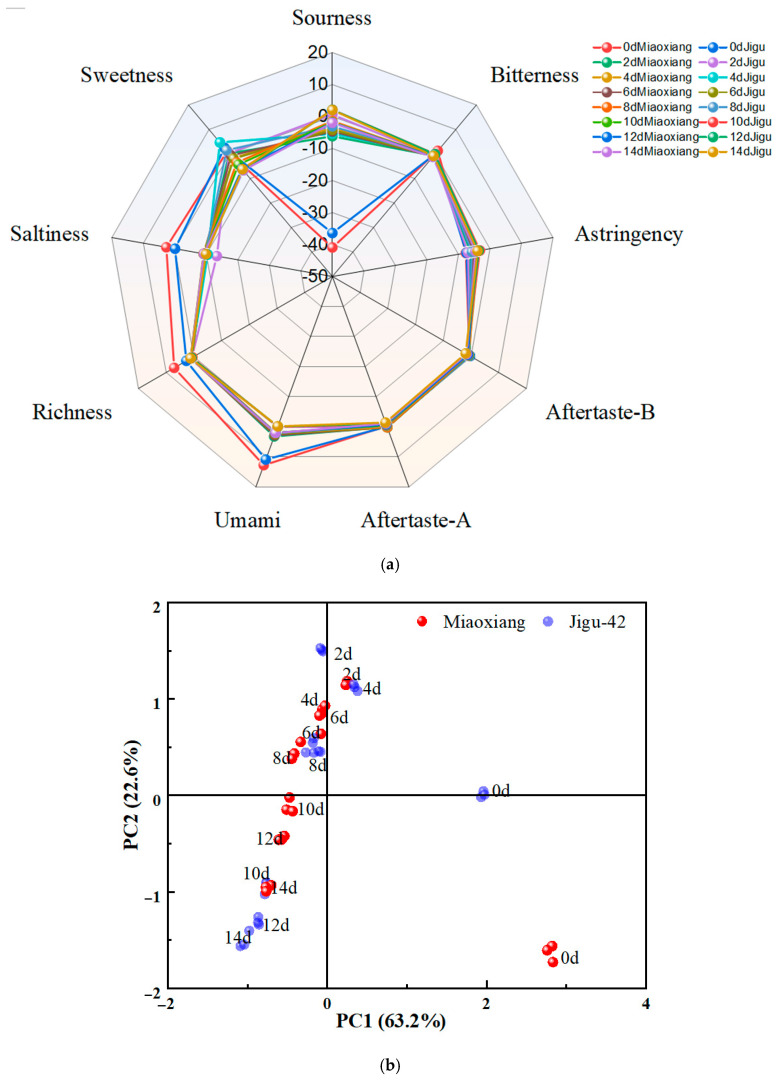

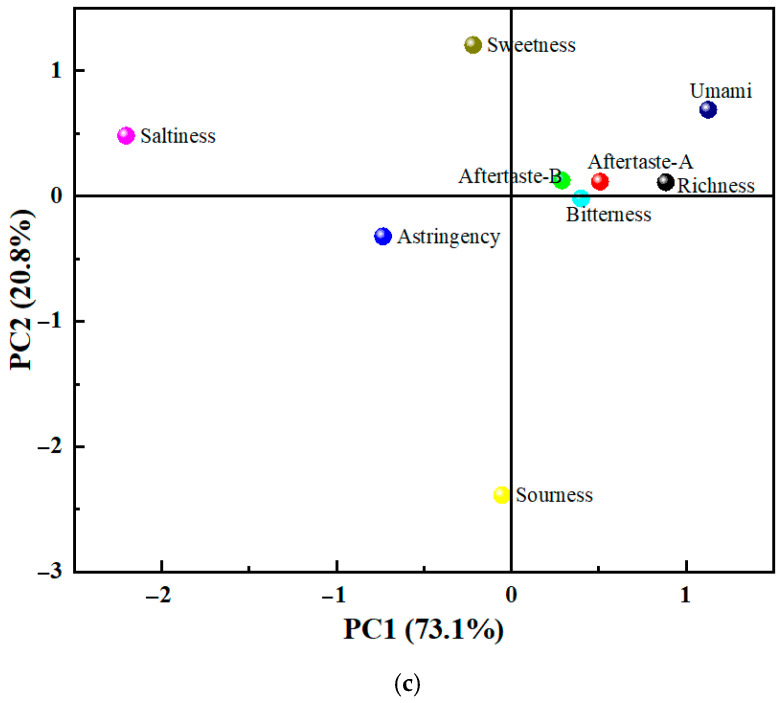
Flavor analysis of different millet varieties: (**a**) the radar image of taste determination using the E-tongue; (**b**) PCA score scatter plot; and (**c**) biplot of sensory attributes via PCA.

Over 700 VCs were identified in “*Red Ferment*”-fermented millet, with only 27 demonstrating significant contributions to aroma perception (odor activity value > 1). E-tongue analysis (Figure 3) showed no significant differences between Miao Xiang glutinous millet and Jigu-42 groups in bitterness, astringency, sourness, aftertaste-astringency (aftertaste-A), or aftertaste-bitterness (aftertaste-B). However, statistically significant variations (*p* < 0.05) were detected in sweetness, saltiness, richness, and umami. During fermentation, sweetness initially increased before declining, while saltiness, richness, and umami progressively decreased. These results demonstrate that taste profile evolution is fermentation time-dependent and primarily driven by the dynamic accumulation of flavor-active metabolites during microbial fermentation.

PCA was performed on the electronic-tongue sensor data to investigate differences in taste characteristics among red fermented-millet varieties. PC1 and PC2 accounted for 63.2% and 22.6% of the total variance, respectively (Figure 3b), indicating that these principal components effectively captured distinct flavor profiles. The unfermented Miao Xiang glutinous millet and Jigu-42 millet varieties showed marked differences compared to fermented samples, demonstrating that fermentation significantly altered millet taste. Samples fermented for 2 to 8 days clustered in the positive PC2 region, while those fermented for 10–14 days were primarily distributed in the Ⅳ quadrant, indicating a clear distinction between early and late fermentation stages.

As shown in Figure 3c, PC1 and PC2 explained 73.1% and 20.8% of the variance, respectively, effectively summarizing the sensor data. Among taste attributes, sweetness, saltiness, and sourness were significantly distinct from other taste attributes.

### 3.5. Correlation Analysis

**Figure 4:**
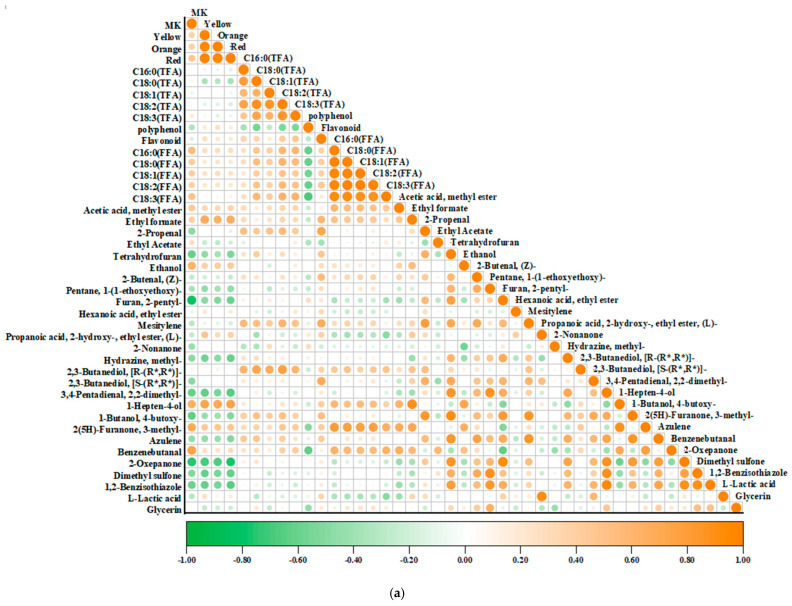

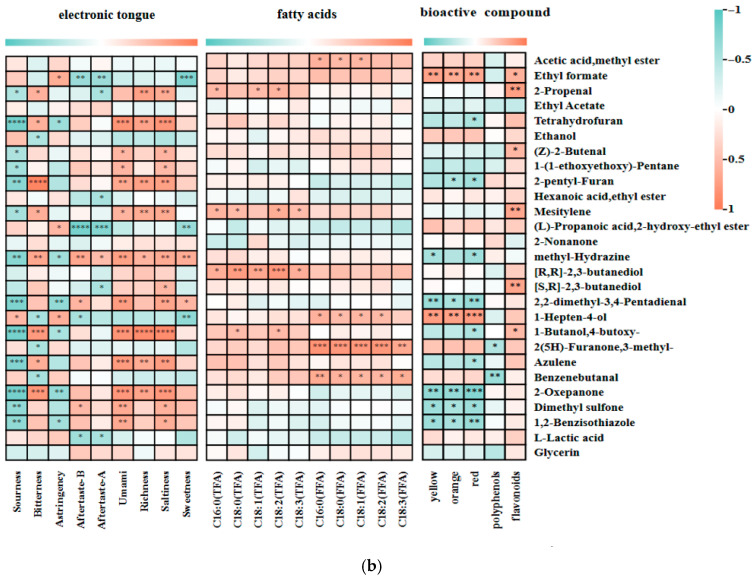
Correlation analysis for the fermented millet: (**a**) correlation heat map of different compounds from red millet; (**b**) correlation heat map between taste profiles, fatty acids, color and volatile compounds. Significant differences are marked on the graph, * (*p* < 0.05), ** (*p* < 0.01), *** (*p* < 0.001), **** (*p* < 0.0001).

Correlation analysis was performed to evaluate interrelationships among the measured parameters. As shown in Figure 4a, MK exhibited significant positive correlations with benzenebutanal (*r* = 0.74, *p* < 0.01) and 3-methyl-2(5H)-furanone (*r* = 0.68, *p* < 0.01), and significant negative correlations with 2-pentylfuran (*r* = −0.82, *p* < 0.01) and ε-caprolactone (*r* = −0.75, *p* < 0.01).

Similarly, the yellow pigment showed highly significant positive correlations with the orange pigment (*r* = 0.96, *p* < 0.001), red pigment (*r* = 0.99, *p* < 0.001), and 1-hepten-4-ol (*r* = 0.74, *p* < 0.01), and highly significant negative correlations with ε-caprolactone (*r* = −0.69, *p* < 0.01), 1,2-benzisothiazole (*r* = −0.61, *p* < 0.05), and 2,2-dimethyl-3,4-pentadienal (*r* = −0.63, *p* < 0.05). The orange pigment showed significant correlations with the red pigment (*r* = 0.97, *p* < 0.001), ethyl formate (*r* = 0.63, *p* < 0.05), 1-hepten-4-ol (*r* = 0.71, *p* < 0.01), and ε-caprolactone (*r* = −0.67, *p* < 0.01). The red pigment exhibited highly significant positive correlations with ethyl formate (*r* = 0.66, *p* < 0.01) and 1-hepten-4-ol (*r* = 0.74, *p* < 0.01), and highly significant negative correlations with 2,2-dimethyl-3,4-pentadienal (*r* = −0.66, *p* < 0.01), ε-caprolactone (*r* = −0.76, *p* < 0.01), and 1,2-benzisothiazole (*r* = −0.65, *p* < 0.01).

Among the measured total fatty acids, palmitic acid (C16:0) showed relatively low correlation with linolenic acid (C18:3), whereas other fatty acids exhibited significant positive intercorrelations. These fatty acids also demonstrated highly significant positive correlations with [R, R]-2,3-butanediol. The free fatty acids exhibited highly significant positive intercorrelations. Specifically, palmitic acid (C16:0) exhibited highly significant positive correlations with stearic acid (C18:0) (*r* = 0.98, *p* < 0.001), oleic acid (C18:1) (*r* = 0.96, *p* < 0.001), linoleic acid (C18:2) (*r* = 0.96, *p* < 0.001), and linolenic acid (C18:3) (*r* = 0.92, *p* < 0.001). Furthermore, all five FFAs showed significant positive correlations with 3-methyl-2(5H)-furanone. Total polyphenols exhibited significant negative correlations with all five free fatty acids (*p* < 0.05), whereas flavonoids demonstrated positive yet relatively weak correlations with both total and free fatty acid fractions (*r* = 0.12–0.35, *p* > 0.05).

As shown in Figure 4b, the flavor substances 4-butoxy-1-butanol, 2-oxepanone, azulene, and tetrahydrofuran showed significant negative correlations with sourness but positive correlations with umami, richness, and saltiness, respectively. Bitterness was significantly positively correlated with 2-oxepanone, 4-butoxy-1-butanol, methylhydrazine, and 2-pentylfuran. (L)-Propanoic acid 2-hydroxy-ethyl ester showed significant negative correlations with aftertaste-A and aftertaste-B.

## 4. Discussion

Millet’s bioactive compounds and VCs could be significantly enhanced by “*Red Ferment*” fermentation, and supplemented with MPs and MK secondary metabolites. Rich in protein, dietary fiber, vitamins, and minerals, millet serves as an excellent source of the carbon, nitrogen, and minerals essential for microbial growth, thereby facilitating the utilization and valorization of its byproducts. Additionally, millet contains high levels of micronutrients, such as iron, magnesium, and phosphorus, that contribute to cellular metabolism and microbial proliferation [33].

*Rhodotorula ruber*, a red yeast species, has emerged as a promising microorganism for industrial applications due to its ability to synthesize valuable metabolites, including carotenoids, enzymes [34], and lipids [35]. Carotenoids, which impart visible yellow, orange, or red pigmentation, are widely utilized in the food, pharmaceutical, nutraceutical, and aquaculture feed additive industries [36]. They also serve as key precursors for aroma compounds [31,37]. The lipids produced by this yeast predominantly consist of unsaturated fatty acids—palmitic (C16:0), stearic (C18:0), oleic (C18:1), linoleic (C18:2), linolenic (C18:3), and myristic (C14:0)—making the products of the reaction suitable for food and pharmaceutical applications [38].

In contrast to *Rhodotorula ruber*, *Monascus purpureus* is employed in solid-state fermentation across various substrates for biopigment production, including soybean meal powder [39], potato [40], Chinese rice wine wastes [41], different rice varieties [42], broken rice [43], ginger [44], textured soy protein [45], sugarcane bagasse [46], potato pomace [47], and highland barley [22]. Both carotenoids (produced by *Rhodotorula ruber*) and MPs (from *M. purpureus*) exhibit antioxidant, anti-inflammatory, and anticancer properties [17]. During growth, *M. purpureus* utilizes gluten and branched-chain starch as substrates to synthesize the metabolite MK. While branched-chain starch is hydrolyzed during fermentation, straight-chain starch remains unreacted; however, the gluten consumption is notably lower than that of starch [48]. This explains why *Japonica* millet (Jigu-42) exhibited a peak MK content on day 2, followed by a decline resulting from nutrient depletion and enzymatic degradation [48].

Furthermore, microbial metabolism also influences flavor compounds. Alcohols are the end products of the microbial metabolism of glucose and amino acids [49], with pleasant aromas of alcohol and fruity and sweet flavors [50]. In these red fermented-millet samples, alcohols were present in high concentrations, such as ethanol, [R, R]-2,3-butanediol, 1-hepten-4-ol, and [S, R]-2,3-butanediol. This is consistent with the results after the fermentation of *Monascus*-fermented rice [51]. The formation of alcohols is related to the action of *Rhodotorula ruber* during the fermentation process. The results showed that the ethanol content increases gradually with the increase in fermentation time. The increase in ethanol content would stimulate the respiratory activity and metabolic growth of the microorganisms involved in the fermentation, increasing the ability of *Monascus* to produce yellow pigment [52], which is in line with the results of our study (Figure 1a and Figure 4b). At the same time, the ability to produce alcohol indirectly indicates the ability to utilize starch, with higher alcohol production leading to better starch utilization [53].

Esters are known for their distinctive flavor and aroma properties, often exhibiting strong fruity notes. These compounds provide a pleasing and complex dimension to numerous foods and beverages, thereby enhancing the overall sensory experience. Several esters, including methyl acetate, ethyl acetate, ethyl lactate, and ethyl caproate, were detected in the red millet samples. Methyl acetate, with a ‘green’ odor, imparts a relatively fresh flavor. Ethyl acetate is known for its sweetness and fruity aroma, while esters such as ethyl lactate and ethyl caproate contribute ‘fruity’, ‘apple’, and ‘red berry’ flavors.

Aldehydes may be produced by the oxidation of fatty acids in grains and by Maillard reactions during heat treatment [50]. Most aldehydes are oxidation products of unsaturated fatty acids (e.g., linoleic and linolenic acids) [54]. Aldehydes tend to decrease significantly with increasing fermentation time, likely due to the activity of hydroperoxidase-cleaving enzymes and alcohol dehydrogenases, which promote the reaction of hydroperoxides with aldehydes, leading to the consumption of the latter and their conversion into other compounds [55].

Acids are mainly formed by the oxidation of alcohols and aldehydes, imparting to the samples aroma characteristics such as creaminess, sourness, and fattiness [56]. In later stages of fermentation, the levels of lactic acid also showed a gradual increase. As a product of microbial fermentation, the accumulation of lactic acid may negatively affect food production quality [57]. The higher acid levels may be attributed to the rapid growth of the fermentative microorganisms and auxiliary microorganisms cultivated at higher temperatures during the early stages of fermentation. Due to this rapid development, protease and lipase activities were significantly increased, accelerating the hydrolysis of the proteins and fats in millet. The acid content of Jigu-42 was 21.75% higher than that of Miao Xiang glutinous millet, which released more fatty acids after the 8th day of fermentation (Figure 1d).

The detected ε-caprolactone showed significant negative correlations with MK (*r* = −0.75, *p* < 0.01) and yellow (*r* = −0.69, *p* < 0.01), orange (*r* = −0.67, *p* < 0.01), and red pigments (*r* = −0.76, *p* < 0.01). Its late-stage decline (days 8–14) coincided with pigment accumulation, suggesting a possible competitive relationship with MK and the synthesis of MPs, though enzymatic evidence is required to confirm this interaction. Notably, ε-caprolactone correlated positively with bitterness, umami, and saltiness; it may activate TAS2R bitter receptors [58], while umami enhancement could involve glutamate receptor modulation [59].

Fatty acids showed positive correlations with key volatile compounds (VIP > 1.5, PLS-DA model). Fatty acid oxidation generates flavor-active compounds, including aldehydes, alcohols, and ketones [60]. During fermentation, microbial metabolism produces fatty acids and alcohols, which are further esterified to form esters—a process strongly influenced by free fatty acid (FFA) content [61]. *Rhodotorula ruber* as an oleaginous yeast synthesizes phospholipids via the Kennedy pathway, while its extracellular enzymes accelerate the hydrolysis of proteins and lipids in millet, releasing amino acids, FFAs and other flavor precursors. Additionally, polyunsaturated fatty acids (PUFAs) in phospholipids can undergo oxidation to form aldehyde and ketone flavor compounds [62].

4-Butoxy-1-butanol showed a strong anti-correlation with sourness, which may result from pH-dependent degradation of its ether-alcohol moiety, though its structural stability under acidic conditions requires further verification. Its positive correlation with saltiness could be associated with Aspergillus fermentation [63], which would be consistent with studies showing that flavor compounds enhance saltiness perception [64]; however, strain-specific controls are necessary to exclude confounding factors. Finally, the strong positive correlation (*r* = 0.74, *p* < 0.01) between 1-hepten-4-ol and MPs (particularly the red pigment) suggests potential biosynthetic linkage or molecular stabilization.

The growing environment of Miao Xiang glutinous millet features significant diurnal temperature variation and abundant precipitation, which facilitates nutrient accumulation. Consequently, its fatty acid content is substantially higher than that of Jigu-42. 2-Pentylfuran, the most abundant heterocyclic compound with vegetable- and fruit-like aromatic properties, is produced by oxidative degradation of unsaturated fatty acids [49]. In contrast, Jigu-42 grows in northern regions characterized by prolonged sunlight exposure and limited precipitation, leading to substantial polyphenol accumulation, an environmental adaptation mechanism.

## 5. Conclusions

This study investigated the effects of “*Red Ferment*” fermentation on bioactive components and flavor profiles in waxy (Miao Xiang glutinous millet) and non-waxy (Jigu-42) millet. Key findings include the following: Both varieties showed significant increases in MPs and MK after fermentation (*p* < 0.05). For MK production, Miao Xiang glutinous millet required extended fermentation (>6 days), whereas Jigu-42 achieved optimal yields in a shorter time (<4 days). Miao Xiang glutinous millet had a higher fatty acid content, whereas Jigu-42 had a higher total polyphenol content. Among the 27 identified volatile compounds, alcohols (e.g., 2,3-butanediol) were the most abundant, accounting for >80% of the total, and contributing a sweet/fermented flavor. Miao Xiang glutinous millet generated 30% more esters and 15% more aldehydes than Jigu-42, while levels of ketones and acids were lower. In summary, this research supports the development of millet-based *Monascus* products with enhanced nutraceutical value and suggests genotype-specific fermentation strategies for quality optimization. Although electronic-tongue/GC-MS data provided objective taste/aroma metrics, the absence of human sensory testing remains a limitation. Subsequent studies will combine physicochemical analyses with controlled consumer trials following ISO 8589:2007 standards [65] to address this gap. Further studies, including metagenomic analysis and process parameter optimization, are needed to elucidate strain–substrate interactions and improve the flavor–functionality balance.

## Figures and Tables

**Table 1:** Effect of fermentation time on chromatic characteristics of different varieties of millet.

Fermentation Time/d	L*	a*	b*	w	ΔE
Miao Xiang glutinous millet					
0d-1	91.53 ± 0.07 ^c^	−2.37 ± 0.04 ^k^	24.69 ± 0.68 ^g^	73.79 ± 0.67 ^f^	
2d-1	89.91 ± 0.11 ^f^	1.33 ± 0.19 ^e^	31.88 ± 0.63 ^e^	66.53 ± 0.64 ^h^	8.25 ± 0.07 ^d^
4d-1	89.04 ± 0.11 ^g^	2.84 ± 0.23 ^d^	36.97 ± 0.58 ^d^	61.33 ± 0.6 ^i^	13.58 ± 0.27 ^c^
6d-1	90.43 ± 0.12 ^e^	0.45 ± 0.07 ^f^	28.53 ± 0.57 ^f^	69.9 ± 0.58 ^g^	4.90 ± 0.1 ^e^
8d-1	83.74 ± 0.56 ^i^	14.41 ± 1.18 ^b^	47.03 ± 0.44 ^c^	48.19 ± 0.86 ^j^	29.02 ± 0.92 ^b^
10d-1	84.00 ± 0.16 ^hi^	13.92 ± 0.43 ^b^	47.13 ± 0.25 ^c^	48.32 ± 0.31 ^j^	28.74 ± 0.82 ^b^
12d-1	84.27 ± 0.10 ^h^	13.08 ± 0.22 ^c^	48.29 ± 0.08 ^b^	47.55 ± 0.09 ^j^	29.13 ± 0.42 ^b^
14d-1	81.18 ± 0.08 ^j^	19.32 ± 0.23 ^a^	52.17 ± 0.04 ^a^	41.27 ± 0.07 ^k^	36.52 ± 0.37 ^a^
Jigu-42					
0d-2	91.80 ± 0.01 ^bc^	−2.25 ± 0.04 ^jk^	21.94 ± 0.03 ^j^	76.47 ± 0.03 ^c^	
2d-2	92.13 ± 0.03 ^a^	−1.64 ± 0.03 ^ij^	17.34 ± 0.19 ^m^	80.89 ± 0.18 ^a^	4.65 ± 0.18 ^e^
4d-2	91.92 ± 0.01 ^ab^	−1.28 ± 0.01 ^hi^	18.45 ± 0.06 ^l^	79.82 ± 0.06 ^b^	3.63 ± 0.07 ^f^
6d-2	91.51 ± 0.02 ^c^	−0.77 ± 0.02 ^gh^	21.15 ± 0.13 ^k^	77.2 ± 0.12 ^c^	1.71 ± 0.03 ^h^
8d-2	91.12 ± 0.02 ^d^	−0.20 ± 0.04 ^fg^	23.65 ± 0.11 ^hi^	74.74 ± 0.11 ^de^	2.75 ± 0.15 ^g^
10d-2	91.49 ± 0.02 ^c^	−0.64 ± 0.04 ^gh^	21.13 ± 0.11 ^k^	77.21 ± 0.11 ^c^	1.83 ± 0.01 ^h^
12d-2	90.99 ± 0.01 ^d^	0.19 ± 0.03 ^f^	24.01 ± 0.04 ^gh^	74.35 ± 0.04 ^ef^	3.3 ± 0.09 ^fg^
14d-2	91.12 ± 0.01 ^d^	0.05 ± 0.03 ^f^	23.02 ± 0.04 ^i^	75.33 ± 0.04 ^d^	2.62 ± 0.09 ^g^

Note: Values are expressed as mean ± standard deviation of measurements in triplicate. Means with different superscript letters (a–m) in the same column differ significantly (*p* < 0.05, one-way ANOVA with Duncan’s multiple range test).

## Data Availability

The original contributions presented in the study are included in the article/Supplementary Material, further inquiries can be directed to the corresponding author.

## Supplementary Materials

The following supporting information can be downloaded at: https://www.mdpi.com/article/10.3390/foods14152692/s1, Table S1: Details of volatile compounds between Miao Xiang glutinous millet and Jigu-42.
