# Supplementary material for: Comparative Analysis of Bioactive Compounds and Flavor Characteristics in Red Fermentation of Waxy and Non-Waxy Millet Varieties
Source: Foods. 2025 Jul 30;14(15):2692. doi: 10.3390/foods14152692 (PMC12346767; doi:10.3390/foods14152692)
Supplement: Supplementary file 1 [file foods-14-02692-s001.zip › foods-3764689-supplementary.pdf]

Table S1. Details of volatile compounds between Miao Xiang glutinous millet and Jigu-42

No.	Compounds	Cas	Samples																Odor
			0d-1	0d-2	2d-1	2d-2	4d-1	4d-2	6d-1	6d-2	8d-1	8d-2	10d-1	10d-2	12d-1	12d-2	14d-1	14d-2	
1	Acetic acid, methyl ester	79-20-9	1.71	0.00	0.56	0.00	1.89	3.60	3.69	1.12	3.63	1.49	2.25	1.64	2.85	0.00	3.02	1.27	estry, fruity, winey, cognac and rum
2	Ethyl formate	109-94-4	12.20	7.69	4.61	2.91	14.72	15.46	26.67	13.22	36.89	12.26	25.98	31.21	55.80	43.77	51.87	23.67	strong, ethereal-fruity, like rum
3	2-Propenal	107-02-8	5.15	2.69	0.13	1.25	0.51	2.02	2.03	1.39	0.83	0.98	2.02	2.14	2.60	2.76	3.31	3.27	fruity
4	Ethyl Acetate	141-78-6	0.87	1.25	93.51	46.20	885.35	124.98	182.29	47.20	82.46	56.86	37.88	26.54	57.04	42.15	52.08	27.22	Ethereal, fruity-grape, sweet, rum-like
5	Tetrahydrofuran	109-99-9	31.06	39.02	4.70	12.22	0.21	13.75	1.67	7.06	6.52	1.76	4.74	3.49	12.51	12.95	16.41	6.75	Ether odor
6	Ethanol	64-17-5	56.28	389.10	580.42	2925.53	1014.98	2334.05	2404.02	1460.60	2199.26	0.00	1845.22	174.12	2279.25	2484.62	4094.70	1379.01	strong alcoholic ethereal medical

7	2-Butenal, (Z)-	15798-64-8	4.55	8.30	0.00	0.00	4.37	5.58	5.45	2.09	3.95	1.20	3.22	2.36	4.15	3.62	4.78	1.94	--
8	Pentane, 1-(1-ethoxyethoxy)-	13442-89-2	0.00	2.62	0.00	0.00	0.31	0.00	0.47	0.05	0.28	0.07	0.21	0.06	0.26	0.00	0.09	0.00	--
9	Furan, 2-pentyl-	3777-69-3	2.75	2.27	0.19	1.05	0.19	1.26	0.21	0.45	0.28	0.44	0.15	1.37	0.24	1.71	0.00	0.32	Fruity,green, earthy beany with vegetable like nuances
10	Hexanoic acid, ethyl ester	123-66-0	1.34	0.00	0.29	1.05	1.10	1.16	0.65	0.47	1.66	0.23	0.59	0.49	0.61	0.70	0.69	11.18	strong, sweet-ethereal like pineapple, with nuances of banana and strawberry
11	Mesitylene	108-67-8	1.74	1.18	0.25	0.44	0.47	1.17	0.84	0.53	0.94	0.00	0.80	0.80	1.01	1.06	1.19	0.54	--
12	Propanoic acid, 2-hydroxy-,	687-47-8	4.92	10.71	1.16	1.60	1.68	2.31	3.69	11.87	5.69	19.00	7.47	63.92	13.94	88.86	23.45	65.34	sour,fruity

	ethyl ester, (L)-																	
13	2-Nonanone	821-55-6	0.54	0.15	0.00	0.00	0.00	0.00	0.12	0.08	0.12	1.31	0.15	0.37	0.00	0.19	0.00	0.25	fresh sweet,Fruity, weedy, earthy, herba
14	Hydrazine, methyl-	60-34-4	32.04	33.02	11.02	17.03	7.80	25.29	11.42	11.67	15.07	7.64	13.84	21.12	13.40	0.00	0.00	0.13	--
15	2,3-Butanediol, [R-(R*,R*)]-	24347-58-8	12.59	0.01	0.01	0.04	2.70	0.02	0.00	0.01	0.00	0.00	2.17	0.00	18.64	0.00	8.61	0.01	--
16	2,3-Butanediol, [S-(R*,R*)]-	19132-06-0	12.21	19.34	2.05	4.44	1.70	4.52	3.37	2.22	7.27	5.31	7.56	12.56	9.31	16.50	13.15	8.72	--
17	3,4-Pentadienal, 2,2-dimethyl-	4058-51-9	0.20	0.64	0.02	0.12	0.00	0.15	0.00	0.00	0.00	0.00	0.00	0.00	0.00	0.00	0.00	0.00	--
18	1-Hepten-4-ol	3521-91-3	0.00	1.14	3.21	24.19	21.87	31.47	56.54	27.06	77.53	20.23	60.32	47.49	77.36	49.78	107.77	34.96	--
19	1-Butanol, 4-butoxy-	4161-24-4	20.11	15.28	3.16	6.56	3.68	8.25	4.78	3.89	5.59	2.39	5.25	6.38	7.00	8.51	9.88	6.70	Ether odor
20	2(5H)-Furanone, 3-methyl-	22122-36-7	0.50	0.00	0.05	0.35	6.48	0.38	40.75	0.00	47.23	1.12	39.83	2.82	44.83	1.21	69.85	0.93	--

21	Azulene	275-51-4	0.35	0.38	0.19	0.19	0.19	0.30	0.22	0.19	0.21	0.10	0.20	0.22	0.19	0.23	0.28	0.13	--
22	Benzenebutanal	18328-11-5	0.36	0.46	1.67	0.67	6.34	1.98	4.50	1.06	4.19	0.61	3.95	1.38	4.86	1.56	5.69	0.97	--
23	2-Oxepanone	502-44-3	0.19	0.23	0.04	0.07	0.04	0.09	0.00	0.00	0.00	0.00	0.00	0.06	0.00	0.09	0.00	0.00	-
24	Dimethyl sulfone	67-71-0	0.24	1.41	0.00	0.00	0.58	0.34	0.00	0.00	0.00	0.00	0.00	0.00	0.00	0.00	0.00	0.00	sulfurous burnt
25	1,2-Benzisothiazole	272-16-2	3.00	8.49	2.53	2.31	1.91	2.95	0.89	0.62	0.97	0.93	1.24	2.49	1.00	2.35	1.88	1.07	--
26	L-Lactic acid	79-33-4	46.62	61.40	0.00	7.28	0.00	3.00	0.00	2.32	0.00	5.30	12.35	263.46	15.65	520.96	20.36	159.92	odorless, sour
27	Glycerin	56-81-5	0.22	7.23	5.55	0.78	2.45	2.46	3.41	3.38	3.80	0.65	1.49	0.00	1.95	1.78	4.77	1.06	odorless, sweet
